# Combination Low-Dose Tissue-Type Plasminogen Activator Plus Annexin A2 for Improving Thrombolytic Stroke Therapy

**DOI:** 10.3389/fncel.2015.00397

**Published:** 2015-10-14

**Authors:** Yinghua Jiang, Xiang Fan, Zhanyang Yu, Zhengbu Liao, Xiao-Shu Wang, Klaus van Leyen, Xiaochuan Sun, Eng H. Lo, Xiaoying Wang

**Affiliations:** ^1^Department of Neurosurgery, The First Affiliated Hospital, Chongqing Medical University, Chongqing, China; ^2^Neuroscience Program, Neuroprotection Research Laboratory, Department of Neurology and Radiology, Massachusetts General Hospital, Harvard Medical School, Boston, MA, USA; ^3^Research Institute of Traditional Chinese Medicine, Tianjin University of Traditional Chinese Medicine, Tianjin, China

**Keywords:** cerebral ischemia, tissue-type plasminogen activator, annexin A2, thrombolysis, combination therapy, focal embolic stroke model, rats

## Abstract

Risk of hemorrhagic transformation, incomplete reperfusion, neurotoxicity, and a short treatment time window comprises major challenges for tissue plasminogen activator (tPA) thrombolytic stroke therapy. Improving tPA therapy has become one of the highest priorities in the stroke field. This mini review article focuses on our recent efforts aimed at evaluating a novel combination approach of low-dose tPA plus recombinant annexin A2 (rA2, a tPA, and plasminogen co-receptor), which might enhance tPA thrombolytic efficacy, while reducing its associated complications related to intracerebral hemorrhagic transformation. Results of our experimental studies using a focal embolic stroke model in rats support the feasibility of the combination approach and suggest the potential for successful clinical translation.

## Limitations of tPA Thrombolytic Stroke Therapy

By stimulating thrombolysis and rescuing the ischemic brain via restoring blood flow, intravenous administration of recombinant tissue plasminogen activator (tPA) remains the most effective intervention with FDA approval for emergency treatment of stroke (Whiteley et al., 2014). However, a short treatment time window, hemorrhagic transformation, poor thrombolytic perfusion rate, and neurotoxicity comprise the major limitations to its application (Alexandrov and Grotta, 2002; Bambauer et al., 2006; Weintraub, 2006). Although other thrombolytic agents are being tested, none has been established as effective or as a replacement for tPA (Wang et al., 2004, 2008; Adams et al., 2007).

Recent clinical investigations have suggested the potential for improving tPA therapy. For example, perfusion-/diffusion-weighted imaging (PWI/DWI) mismatch and the diffusion/fluid attenuated inversion recovery (DWI/FLAIR) mismatch might have implications in selecting patients for reperfusion therapy (Shah et al., 2015; Wouters et al., 2015). A randomized phase III trial, ECASS III, designed to test treatment with tPA at 3–4.5 h, showed improved clinical outcomes for ischemic patients with thrombolytic treatment (Hacke et al., 2008). However, there was a detectable large difference in odds-ratios between early reperfusion (approximately 2.8 OR) and delayed reperfusion (approximately 1.4 OR). The benefits of thrombolysis are, thus, still heavily dependent on the treatment time, and tPA remains associated with increased risk of intracranial hemorrhage and reperfusion injury (Hacke et al., 2008). It has been clinically prioritized to seek combination therapies that may extend the therapeutic window, reduce tPA-associated hemorrhagic transformation, and improve thrombolytic efficacy (Wang et al., 2004, 2008; Whiteley et al., 2014).

## Pleiotropic Effects of Exogenous tPA for Stroke Reperfusion Therapy

Ischemic stroke is a thrombotic cerebrovascular event. For emergency treatment, intravenous tPA administration is intended to reopen occluded vessels by lysis of the thrombus, thereby improving clinical outcome through restoration of regional cerebral blood flow (CBF), thus salvaging the ischemic brain tissues (Fugate and Rabinstein, 2014). One limitation is that tPA thrombolysis is only partially or fully successful for re-canalization in about 50% of patients (Hacke et al., 2004). How does the ischemic brain respond to tPA in the non-responders within the context of weakened vessels and perturbed neurovascular homeostasis? Although unequivocal human data are lacking, experimental investigations in animal models suggest that tPA thrombolytic stroke therapy may have deleterious consequences due to the non-thrombolytic actions of tPA (Kaur et al., 2004). More investigational efforts are needed to dissect the molecular signaling mechanisms initiated by the infused exogenous tPA in the occluded vessel and ischemic brain. Accumulating experimental data suggest that exogenous tPA may have additional pleiotropic actions within the brain (Kaur et al., 2004), such as direct vasoactivity (Nassar et al., 2004; Armstead et al., 2009) enhanced excitotoxicity (Nicole et al., 2001), and activation of extracellular proteases (Nicole et al., 2001; Wang et al., 2003, 2004; Benchenane et al., 2004; Zeevi et al., 2007). These non-thrombolytic actions of tPA may exacerbate edema, increase ischemic neurotoxicity, damage the blood–brain barrier, and increase risk of cerebral hemorrhage, ultimately compromising its usefulness as a thrombolytic agent (Yepes et al., 2003, 2009; Su et al., 2008; Armstead et al., 2009).

Re-canalization has been well established as an important predictor of better stroke outcome, regardless of thrombolytic modality employed. However, a major remaining challenge is that exogenous tPA may potentiate ischemia-induced blood–brain-barrier disruption, increase the risk of symptomatic intracranial hemorrhage, which restricts prolonging the therapeutic time window (Tsivgoulis et al., 2014). One strategy may overcome these dose-dependent side effects of tPA by simply lowering the tPA dose, but this step would likely reduce the perfusion efficacy. Clearly, an optimization strategy for tPA thrombolytic therapy requires rebalancing the potential benefits of reperfusion against the detrimental effects of exogenous tPA (Wang et al., 2004, 2008).

## tPA Receptor Annexin A2 and Fibrinolytic Assembly

In fibrinolysis, tPA plays a key role by enzymatically converting clot-bound plasminogen to active plasmin, which degrades cross-linked fibrin to break down fibrin-containing thrombi. This process is called fibrinogenolysis (Ranby and Brandstrom, 1988). Interestingly, recent vascular biology studies have demonstrated that tPA may interact with cellular receptors to activate specific signal transduction pathways (Wang et al., 2008). A new concept of fibrinolytic assembly for cell-surface fibrinolysis was proposed, in which the tPA conversion of plasminogen to active plasmin is precisely orchestrated through a multi-molecular complex, consisting of tPA, the annexin A2 heterotetramer, and plasminogen (Kim and Hajjar, 2002).

Annexin A2 is a 36-kDa cell-surface protein, a calcium-dependent phospholipid-binding protein. In complex with its binding partner p11, annexin A2 forms a heterotetrameric (A2_2_p11_2_) receptor for both plasminogen, the inactive precursor of plasmin, and its activator, tPA (Birnbaum et al., 1999). The assembled complex of tPA–annexin A2–­plasminogen increases the catalytic efficiency of tPA in converting plasminogen to plasmin about 60-fold compared with the same amount of tPA alone (Hajjar and Menell, 1997; Birnbaum et al., 1999; Kim and Hajjar, 2002). More experimental evidence suggest that fibrinolytic assembly plays a critical role in maintaining blood and vascular homeostasis (Hajjar and Acharya, 2000). Additionally, annexin A2 exists in both membrane-bound and soluble forms of vascular endothelial cells (Siever and Erickson, 1997), and it can be transported to the cell surface in response to cellular stress (Deora et al., 2004). Complete deficiency of annexin A2 in mice leads to a loss of tPA cofactor activity, intravascular fibrin accumulation, and failure to clear arterial thrombi. In sum, these experimental findings support an important role of annexin A2 in fibrinolytic assembly (Ling et al., 2004).

## Manipulating tPA Fibrinolytic Assembly for Improving Thrombolytic Stroke Therapy

Biologically, tPA efficiently converts plasminogen to clot-­dissolving plasmin relying on the fibrinolytic assembly of a ­trimetric complex of tPA–annexin A2–plasminogen. However, clinically giving a large amount of tPA alone may lead to inefficient assembly of the tPA–annexin A2–plasminogen complex due to a limiting amount of annexin A2. This in turn would reduce the efficacy of the tPA in converting plasminogen to plasmin, which may be partially responsible for the shortcomings of tPA reperfusion stroke therapy. Thus, “High dose tPA required, high hemorrhage risk, low reperfusion efficiency, and short therapeutic time window,” which is a major challenge in our field, can perhaps be avoided (Lo et al., 2002; Wang et al., 2004). It may revolutionize tPA-based stroke therapy if the tPA fibrinolytic assembly can be enhanced and utilized clinically. Because plasminogen (plasmin precursor) exists in circulation, binds to the endothelial cell surface and is enriched in the clot (Sakharov and Rijken, 1995; Birnbaum et al., 1999; Hajjar and Krishnan, 1999), intravenous tPA combined with annexin A2 will locally form tPA–annexin A2–plasminogen complexes and consequently amplify plasmin generation, resulting in more effective and specific fibrinolysis (Birnbaum et al., 1999). By translating the tPA fibrinolytic assembly into tPA therapy development, we hypothesized that combining recombinant annexin A2 protein (rA2) will lower the required dose of tPA for reperfusion, while enhancing thrombolytic efficacy, and attenuating intracerebral hemorrhagic (ICH) transformation. By doing so, it will prolong therapeutic time windows and improve long-term outcomes (Fan et al., 2010).

## Experimental Investigation of Low-Dose tPA Plus rA2 Combination

In the past few years, we have tested this hypothesis in a rat focal embolic stroke model. Our experimental findings support the feasibility of this approach and suggest clinical translation potential (Fan et al., 2010; Zhu et al., 2010; Walvick et al., 2011; Wang et al., 2014; Jiang et al., 2015). Consistent with previous reports (Cesarman et al., 1994; Kim and Hajjar, 2002), *in vitro* plasmin activity assays showed that rA2 significantly amplified tPA-mediated plasmin generation, and equivalent levels of *in vitro* plasmin activity can be reached by using high-dose tPA alone or lower-dose tPA in combination with rA2 (Zhu et al., 2010). Because of species-related differences in fibrin specificity, the equivalent effective dose of human recombinant tPA in the rat was about 10 times higher than the dose used in humans, or about 10 mg/kg (Korninger and Collen, 1981). In a focal embolic stroke model in rats, when animals were treated intravenously 2 h after initiation of ischemia, the 25–50% lower-dose tPA plus rA2 combination was as effective as the standard high-dose tPA alone in restoring perfusion and reducing infarct size (Zhu et al., 2010). This suggests that rA2 can make low-dose tPA more effective in an embolic stroke animal model. Improved reperfusion by the combination was confirmed by MRI analysis in focal embolic stroke of rats (Walvick et al., 2011). We extended these promising findings by asking whether the benefits of tPA plus rA2 combination therapy can be sustained for long-term neurological outcomes. We compared the effects of intravenous high-dose tPA alone (10 mg/kg) versus a combination of low-dose tPA (5 mg/kg) plus 10 mg/kg rA2 in a model of focal embolic cerebral ischemia in rats treated at 3 h after embolization. Compared with conventional high-dose tPA alone, the combination significantly decreased infarction (19.6% reduction, *P* < 0.05) and considerably improved neurological function (*P* < 0.05) at 1-month after stroke (Wang et al., 2014).

In the most recent experimental study, we asked whether the same dose regimen of the combination therapy is still more efficacious and safer when the treatment time window is delayed to 4 h after stroke (Jiang et al., 2015). Our experimental results showed the combination slightly reduced brain infarction compared to saline (9.2% reduction), and tPA (7.4% reduction) at 24 h after stroke, although the reductions did not reach statistical significance, whereas the combination significantly reduced (22.2% reduction, *P* < 0.05) the conventional tPA-elevated ICH transformation. At 7 days after stroke, the combination significantly diminished the conventional tPA alone-elevated iron deposition in peri-lesion areas (68.2% reduction, *P* < 0.05). At 1 month after stroke, the combination significantly improved sensorimotor function recovery (*P* < 0.05) accompanied by a higher microvessel density in the peri-infarct areas compared to rats treated with conventional tPA alone group (*P* < 0.05). Given at a 4-h delay time point after stroke, these experimental results suggest the low-dose tPA plus rA2 therapy combination provides a safer profile by lowering the risk of ICH transformation, accompanied by an improved neurological function recovery after stroke (Jiang et al., 2015). Clinically even within the 3-h time window, intravenous tPA only results in partial or complete reperfusion to about 50% stroke patients. Unfortunately, we are still unable to predict who are the tPA thrombolysis responders or non-responders before giving tPA to ischemic stroke patients. The 50% non-responders may face a higher risk of tPA thrombolytic therapy-associated ICH transformation, where the 3-month mortality was about 60% for ischemic stroke patients who had ICH after receiving intravenous tPA administration (Hacke et al., 2004). Therefore, the notably lower hemorrhagic transformation by the combination treatment might reduce mortality and improve long-term outcome clinically. Regarding the underlying molecular mechanisms of the better neurological function recovery by the delayed combination treatment, we might speculate that in addition to the reduction in tPA-associated ICH transformation, the lower tPA dose and resulting rA2–tPA complex might limit tPA brain penetration-associated neuronal excitotoxicity (Yepes et al., 2009), and that rA2 might bind and neutralize angiostatin-associated endothelial toxicity (Tuszynski et al., 2002), where angiostatin is one of the tPA–plasminogen converting products. Ultimately, the decreased hemorrhagic brain damage and fewer neurovascular side effects might translate into better vascular remodeling and improvement of long-term neurological outcome (Fan et al., 2010). We acknowledge that both tPA therapy-mediated hemorrhagic risk and functional recovery involves complex cascades of BBB, neurovascular and gliovascular responses (Jiang et al., 2015), and the full spectrum of these associated molecular mechanisms remains to be elucidated (Zhang and Chopp, 2009). Some of our ongoing experiments are aiming to address these questions.

Since annexin A2 accelerates the activation of plasmin by complexing with tPA and plasminogen, and this complex binds to the endothelial cell surface and is enriched in the clot (Sakharov and Rijken, 1995; Hajjar and Krishnan, 1999), the rA2–tPA combination may thus generate more plasmin locally at the clot site. Alternatively, within the thrombus, the fibrin-associated plasminogen could be activated by A2-associated tPA, resulting in more effective fibrinolysis. In addition, rA2-bound tPA and plasmin might be relatively protected from their circulating inhibitors, plasminogen activator inhibitor-1 (PAI-1), and alpha_2_-antiplasmin (alpha_2_-AP) (Fan et al., 2010). Although the underlying molecular mechanisms for the improved therapeutic efficacy by the combination needs to be further elucidated, from a clinical perspective, all these possible mechanisms may ultimately yield a rebalancing of tPA thrombolytic reperfusion benefits against its detrimental side effects.

## Summary

**Figure 1:**
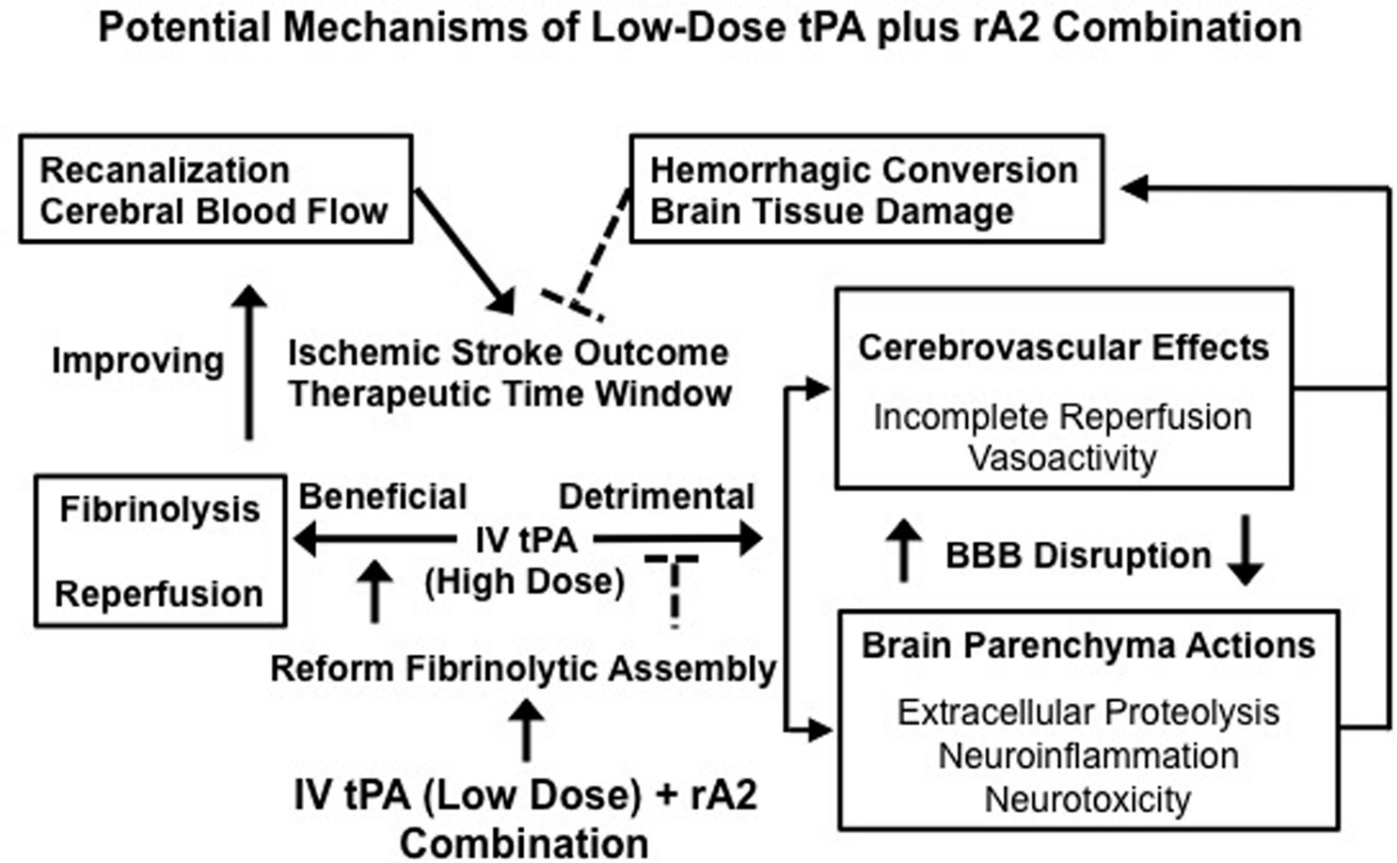
**Changing the balance of thrombolytic reperfusion benefits versus hemorrhagic side effects of exogenous tPA through the use of a low-dose tPA plus rA2 combination**. A schematic outline for the potential mechanisms of the combination is to link multiple beneficial and detrimental effects of exogenous tPA acting intravascularly and extravascularly. By rebalancing the beneficial/detrimental effect ratio, our new combination approach may improve both therapeutic efficacy and safety of tPA-based thrombolytic stroke therapy.

Our experimental investigations provide strong evidence in support of the hypothesis that the combination of low-dose tPA plus rA2 improves stroke thrombolytic therapy. By improving the fibrinolytic assembly to accelerate plasmin generation, thus enhancing thrombolytic reperfusion efficacy, the combination restores CBF, and rescues ischemic brain tissue more efficiently. This lowers the required dose of tPA, which minimizes both tPA-direct and -associated side effects of neurotoxicity, neuroinflammation, extracellular proteolytic dysfunction, and hemorrhagic conversion (Fan et al., 2010). Changing the rebalance by improving reperfusion benefits while reducing side effects through the combination may diminish the risk of ICH, prolong the therapeutic time window, and improve long-term outcome of ischemic stroke patients (Figure 1). We acknowledge that safety issues and all translational aspects of this potential treatment need to be carefully investigated for future preclinical evaluation.

## Conflict of Interest Statement

The authors declare that the research was conducted in the absence of any commercial or financial relationships that could be construed as a potential conflict of interest.
